# *QuickStats: *Prevalence of Obesity* and Severe Obesity^†^ Among Persons Aged 2–19 Years — United States, 1999–2000 Through 2021–2023^§^

**DOI:** 10.15585/mmwr.mm7341a5

**Published:** 2024-10-17

**Figure:**
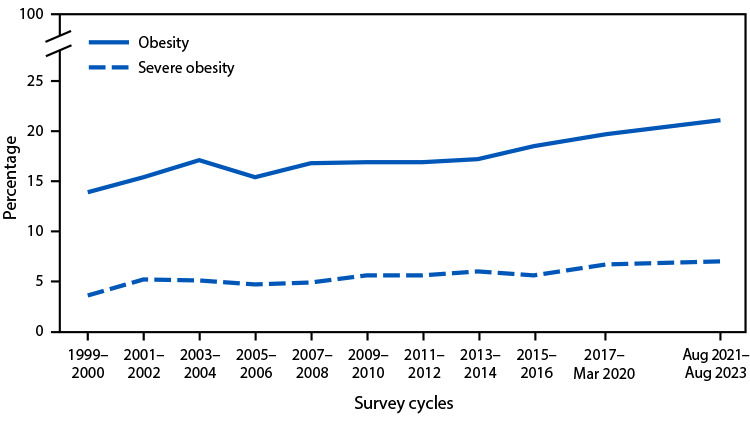
From 1999–2000 through August 2021–August 2023, the prevalence of obesity among persons in the United States aged 2–19 years increased from 13.9% to 21.1%, and the prevalence of severe obesity increased from 3.6% to 7.0%.

**Supplementary Table:** https://stacks.cdc.gov/view/cdc/164014

**Source:** National Center for Health Statistics, National Health and Nutrition Examination Survey, 1999–2000 through August 2021–August 2023. https://www.cdc.gov/nchs/nhanes/index.htm

For more information on this topic, CDC recommends the following link: https://www.cdc.gov/obesity/family-action/index.html.

